# Analysis of Exosomal miRNA and Hepatic mRNA Expression in the Dysregulation of Insulin Action in Perimenopausal Mice

**DOI:** 10.1155/jdr/6251747

**Published:** 2025-01-09

**Authors:** Yu Yang, Yu Chen, Changju Liu, Su Wang, Yijing Zhao, Wen Cao, Kun Wang

**Affiliations:** ^1^Department of Endocrinology, The Affiliated Jiangning Hospital of Nanjing Medical University, Nanjing, Jiangsu Province, China; ^2^Department of Endocrinology, Affiliated Hospital of Integrated Traditional Chinese and Western Medicine, Nanjing University of Traditional Chinese Medicine, Nanjing, Jiangsu Province, China

**Keywords:** hepatic mRNAs, insulin resistance, insulin signaling, perimenopause, plasma exosomal miRNAs

## Abstract

**Introduction:** The aim of present study was to evaluate the impact of perimenopause on insulin resistance. Specifically, insulin sensitivity was assessed in a perimenopausal mouse model treated with 4-vinylcyclohexene diepoxide (VCD), together with the changes in exosomal miRNA and hepatic mRNA expression profiles.

**Methods:** Homeostasis model assessment of insulin resistance (HOMA-IR) was utilized to assess the status of insulin resistance, and insulin action was evaluated during menopausal transition. RNA sequencing (RNA-seq) analysis was used to identify altered expression profiles of exosomal miRNAs and hepatic mRNAs. Differentially expressed miRNA (DEM)–differentially expressed gene (DEG) network analyses were also conducted. Furthermore, altered expression levels of these exosomal miRNAs and genes were validated in plasma exosomes and liver tissue of perimenopausal mice.

**Results:** HOMA-IR in VCD-treated mice was significantly increased, and hepatic glycogen was significantly decreased. Key exosomal miRNAs (miR-17-3p, miR-134-5p, miR-700-5p, and miR-6899-3p) and hepatic genes (*G6pdx*, *Ptpn2*, *Lepr*, *Kras*, and *Braf*) may be associated with impaired insulin signaling during perimenopause.

**Conclusion:** The perimenopausal period acts as a potential factor in introducing insulin resistance as evidenced by impaired insulin action and altered expression profiles of exosomal miRNAs and hepatic genes. The present study contributes to the understanding that abnormal cargos carried by plasma exosomes, such as miRNAs, may be related to altered expression of the corresponding genes in the liver and abnormal insulin response.

## 1. Introduction

Perimenopause refers to the transitional period leading up to menopause, marking the end of the reproductive years [1, 2]. According to the World Health Organization (WHO), women aged 50 years and older accounted for 26% of all women and girls worldwide in 2021, which was up from 22% 10 years earlier. Women experience hormonal changes during the transition to menopause, including increases in serum follicle-stimulating hormone (FSH) levels and decreases in estradiol (E2) levels [3]. Due to changes in hormone levels, perimenopause leads to several health risks, including an increased risk of heart disease, osteoporosis, and metabolic syndrome [4, 5]. The odds of developing metabolic syndrome are the highest during perimenopause independent of age. Cross-sectional studies have suggested that the prevalence of metabolic syndrome increases from premenopause to postmenopause in women independent of age [6, 7]. Growing evidence shows that metabolic disorders, such as insulin resistance, are critical factors that can worsen symptoms of perimenopause and menopause [8–10].

Insulin resistance, which is linked to prediabetes and Type 2 diabetes, is a condition when cells do not respond to insulin well, leading to elevated blood sugar levels. Recent studies have demonstrated that increased incidence of insulin resistance is related to perimenopause in women [8]. However, little is known about the specific molecular mechanisms that cause impaired insulin sensitivity and lead to insulin resistance during perimenopause. The liver plays a central role in the systemic regulation of glucose metabolism and insulin clearance [11]. Normally, insulin attenuates glucose production in the liver by inhibiting enzymes involved in gluconeogenesis [12]. However, aberrant hepatic insulin action is a primary driver of insulin resistance, in which much higher circulating insulin levels are needed to control blood glucose levels [13]. Many studies have focused on the impacts of perimenopause on hepatic glucose output and insulin signaling [14, 15]. Compared to young women, postmenopausal women experience an increase in hepatic glucose production [16]. However, the underlying mechanism by which perimenopause induces hepatic insulin resistance remains unclear.

Exosomes are small vesicles secreted by cells that play a crucial role in intercellular communication by transferring cellular cargoes to recipient cells [17]. Exosomes carry various molecules, including proteins, lipids, mRNA, and other RNA species, such as long noncoding RNA, circular RNA, and microRNA (miRNA) [18]. Among these molecules, miRNAs are the most abundant cargo molecules [19]. As endogenous noncoding RNA molecules, miRNAs are 21–25 nucleotides long, and they regulate various biological processes at the posttranscriptional level. The primary role of miRNAs is to bind to target mRNAs of 3-untranslated regions (UTRs) to repress protein translation or induce cleavage of target mRNAs. Thus, changes in the profile of exosomal miRNAs can be detected in association with diverse diseases, including metabolic disease [20]. The pathogenic role of miRNA and exosomal miRNA has recently become a research hotspot in menopausal transition [21, 22]. Kangas et al. detected aberrant serum exosomal miRNAs during perimenopause [23]. Increasing evidence suggests a crucial role of exosomal miRNAs in insulin resistance and altered glucose metabolism. A previous study has demonstrated that exosomes isolated from obese mice induce glucose intolerance and insulin resistance when administered to adipose tissue of lean mice [24]. In addition, macrophage-derived exosomal miRNAs modulate in vivo and in vitro insulin sensitivity [25]. However, it remains unknown whether exosomal miRNAs participate in the occurrence of insulin resistance in women near menopause.

The aim of the present study was to investigate the effects of perimenopause on the regulation of serum exosomal miRNA expression, the regulation of hepatic mRNA expression, and changes in insulin sensitivity. In the present study, 4-vinylcyclohexene diepoxide (VCD) treatment, which gradually leads to ovarian failure in mice and is hormonally equivalent to human natural conditions, was utilized to generate a C57BL/6 mouse perimenopausal model [26]. The National Toxicology Program has reported that VCD is not toxic to other tissues or organ systems. The duration of perimenopause of women can last as long as several years. However, the perimenopause stage of VCD-treated mice maintains only 17 days, which is too short to induce obvious change in insulin sensitivity. As a result, a short term of high-fat diet (HFD) was utilized to amplify the susceptible effect of perimenopause in insulin resistance [27]. The alterations of plasma exosomal miRNA and hepatic mRNA expression were examined after treatment with VCD and HFD to determine a potential mechanism involved in perimenopause-induced impairment of insulin sensitivity. This study focuses on exosomal miRNAs as potential biomarkers for metabolic changes during perimenopause which could provide new insights into early interventions aimed at preventing insulin resistance and related metabolic disorders in women.

## 2. Materials and Methods

### 2.1. Experimental Animal Models

#### 2.1.1. Animals

In our experiments, we utilized C57BL/6 mice, which are commonly used in metabolic studies due to their well-characterized physiology. Prior to the initiation of the study, specific health-related factors were considered during the selection process. All mice were screened for general health status to ensure they were free from any underlying conditions that could affect metabolic outcomes. This included assessing their body weight, activity levels, and overall physical condition. Furthermore, the mice were acclimatized in the animal facility for 1 week prior to treatment to minimize stress-related variables that could influence experimental results. This careful selection process is crucial for ensuring that the observed effects of VCD treatment and HFD feeding are attributable to the interventions rather than confounding health factors.

Eight-week-old female C57BL/6 mice (*n* = 20) were purchased from the Experimental Animal Center of Nantong University (China) and housed in the Affiliated Hospital of Integrated Traditional Chinese and Western Medicine Animal Facility. All animal experiments were performed under guidelines set by the Animal Care and Use Ethics Committee of Nanjing University of Chinese Medicine. Food and water were provided ad libitum. Mice were kept on a 12:12-h light–dark cycle.

#### 2.1.2. VCD Treatment

After 1 week of acclimation, female mice were randomly divided into the VCD group and control group (10 mice/group). Mice were intraperitoneally (ip) injected with VCD (Sigma–Aldrich, St. Louis, Missouri, United States) at a concentration of 160 mg/kg (VCD group, *n* = 10) or sesame oil (vehicle control (CON) group, *n* = 10) for 20 consecutive days [28]. Mice were fed with a standard diet, consisting of 18% protein and 6% fat. The VCD model closely mimics the development of ovarian failure during the perimenopausal period approximately 35–52 days after the onset of dosing.

#### 2.1.3. Induction of Insulin Resistance

**Figure 1:**
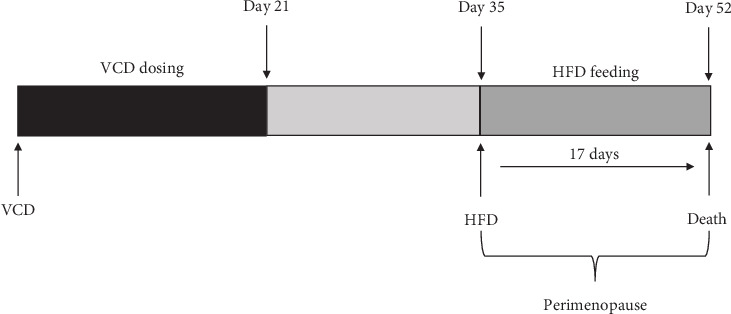
Timeline of the VCD group. Eight-week-old female C57BL/6 mice were intraperitoneally (ip) injected with 4-vinylcyclohexene diepoxide (VCD) to induce gradual ovarian failure. The mice were treated with VCD for 20 consecutive days and with HFD for 17 consecutive days. Large arrows indicate the initial day of treatment with VCD or HFD. Death followed 52 days after VCD injection.

During the transitional period to ovarian failure, mice were initially fed with a standard diet (18% protein and 6% fat) until Day 34 after VCD treatment, and they were then switched to a HFD (16.4% protein and 58% fat) to induce insulin resistance (Figure 1). Mice in the CON and VCD groups received the same feeding pattern.

### 2.2. Evaluation of Serum Hormones and Insulin Resistance

#### 2.2.1. Insulin Tolerance Test (ITT)

At the end of the study, animals were fasted for 4 h and weighed. Blood samples were obtained from the tail veins to determine blood glucose at time zero. All mice received 0.75 U/kg insulin (Wanbang Biopharmaceuticals, China) via ip injection. At 15, 30, 45, and 60 min after insulin administration, blood glucose was measured using an automatic glucometer (Roche, Germany).

#### 2.2.2. Calculation of Homeostasis Model Assessment of Insulin Resistance (HOMA-IR)

HOMA-IR is used to assess whole-body insulin resistance by measuring fasting glucose and insulin concentration. HOMA-IR was calculated using the following formula: HOMA − IR = [glucose concentration (mmol/L) × insulin concentration (*μ*U/L)]/22.5. Blood samples were collected from the tail vein and orbital fossa to determine blood glucose and serum insulin levels. Fasting blood glucose was measured using an automatic glucometer (Roche, Germany), and serum insulin was assayed using an immunoassay kit (Insulin ELISA Kit, EZassay, China).

#### 2.2.3. Serum Hormones

After euthanization, blood samples were collected from the orbital fossa to determine serum hormone levels, and all mice were then sacrificed. Liver, ovaries, and other organs were excised, frozen in liquid nitrogen, and stored at −80°C until use. The serum levels of 17*β*-E2 and FSH were assayed using immunoassay kits (E2 ELISA Kit, Abcam, United Kingdom; FSH ELISA Kit, Abnova, China). The absorbance at 450 nm was determined using a microplate reader (BioTek, United States), and the hormone concentration was calculated by constructing a standard curve by plotting a graph of the absorbance of each reference standard.

### 2.3. Histopathological Evaluation

The liver and ovaries were fixed overnight at 4°C in 4% (*w*/*v*) paraformaldehyde (PFA). Tissues were then embedded in paraffin, sectioned at 5 *μ*m, and stained with hematoxylin and eosin (H&E) for observation using a light microscope.

### 2.4. Glycogen Detection

The hepatic glycogen content was examined by colorimetric assays (Abcam Glycogen Assay Kit, United States) according to the manufacturer's protocol. Paraffin-embedded tissue sections were subjected to periodic acid-Schiff (PAS) staining to detect glycogen in hepatocytes using the PAS Stain Kit (Servicebio, China) according to the manufacturer's instructions. Positive staining was visualized by light microscopy.

### 2.5. Exosome Isolation and Identification

Blood samples were collected into ethylenediaminetetraacetic acid (EDTA) tubes and immediately centrifuged at 1500 rpm for 10 min. Plasma was transferred to a new tube, and exosomes were isolated using the ExoQuick Plasma Prep and Exosome Precipitation Kit (SBI, United States). The purified exosomes were washed once with phosphate-buffered saline (PBS) and resuspended for further characterization.

Exosomes extracted from plasma samples were characterized by Western blot analysis, nanoparticle tracking analysis (NTA), and transmission electron microscopy (TEM). For TEM, isolated exosomes were fixed with glutaraldehyde and placed onto copper mesh formvar-coated grids. The grids were stained with 2% phosphotungstic acid, and the samples were observed using a Hitachi TEM (Hitachi HT7700, Japan). Exosomal markers, including CD63 and TSG101, were evaluated by Western blot analysis to verify the exosomes and compare to parental cells. The size and concentration of exosomes were detected by NTA. For NTA, the samples were diluted with PBS and loaded into the injection tube with a 1-mL syringe. Using the standard operating procedure, the samples were analyzed with a nanoparticle analyzer (NanoSight NS300, United Kingdom).

### 2.6. Library Preparation and Transcriptome Sequencing

Total RNA was extracted from plasma exosomes and liver tissue using TRIzol (Invitrogen, United States). RNA quality was verified using a 2100 bioanalyzer (Agilent, United States) and a ND-2000 (NanoDrop Technologies, United States). The small RNA library was generated using the SMARTer mRNA-Seq Kit (Clontech, United States), and the transcriptome library was generated using the TruSeq RNA Sample Preparation Kit (Illumina, United States). The miRNA and mRNA libraries were sequenced using an Illumina NovaSeq 6000 (Applied Biosystems, United States) at Shanghai Majorbio Bio-Pharm Biotechnology Co. Ltd. (Shanghai, China) according to the manufacturer's instructions.

### 2.7. Analysis of Differentially Expressed miRNAs (DEMs) and Differentially Expressed Genes (DEGs)

The expression level of each miRNA and mRNA was calculated using the transcript per million (TPM) reads method in the cloud platform provided by Shanghai Majorbio Bio-Pharm Biotechnology. Corrected *p* value < 0.05 and fold change > 2.0 were used as thresholds to determine significant DEMs and DEGs using DESeq2 [29].

### 2.8. Integrated Analysis of DEMs and DEGs

To further construct the relationship between the critical DEMs and their target DEGs, TargetScan, miRDB, miRTarBase, and miRWalk were used to predict the candidate target genes of DEMs. The candidate target genes were compared with the DEG results, and they were referred to as differentially expressed target genes (t-DEGs). A Spearman coefficient < −0.6 and *p* value < 0.05 were selected based on the calculation of the expression of DEMs and t-DEGs. Cytoscape v3.10.2 software was used to construct a DEM–DEG regulatory network. Additionally, Gene Ontology (GO) and Kyoto Encyclopedia of Genes and Genomes (KEGG) pathway analyses were used to identify the significantly enriched GO terms and metabolic pathways of the t-DEGs. The *p*‐adjusted < 0.05 threshold value was corrected by the Bonferroni method.

### 2.9. RNA Sequencing (RNA-seq) Data Validation

Exosomal miRNAs were extracted using a miRNeasy Mini Kit (QIAGEN, Germany) according to the manufacturer's instructions. Primers for exosomal miRNAs were designed by RiboBio Co. Ltd. (Guangzhou, China), and reverse transcription was performed using miDETECT A Track (RiboBio, China) according to the manufacturer's instructions. Total RNA was extracted by TRIzol reagent (Invitrogen, United States), and the ReverTra Ace qPCR RT Kit (TOYOBO, Japan) was used for reverse transcription. Quantitative real-time polymerase chain reaction (qRT-PCR) was performed using the Quant Studio Dx System (Life Technology, United States) and SYBR Green Realtime PCR Master Mix (TOYOBO, Japan). The relative expression level for each exosomal miRNA and mRNA was calculated by the 2^−△△CT^ method using cel-miR-39-3p and GAPDH as the internal and external references, respectively. Each sample was run in triplicate. The primers are shown in Table 1.

### 2.10. Western Blot Analysis

Western blot analysis was performed as previously described. Briefly, the harvested exosomes were lysed in radioimmunoprecipitation assay (RIPA) buffer supplemented with protease and phosphatase inhibitors, and protein levels in lysates were quantified using a bicinchoninic acid (BCA) Protein Assay Kit (Beyotime Biotechnology, China). Equal protein quantities (40 *μ*g/lane) were separated by 10% sodium dodecyl sulfate–polyacrylamide gel electrophoresis (SDS-PAGE) and transferred to a polyvinylidene fluoride (PVDF) membrane (Millipore, United States). After blocking with 5% bovine serum albumin (BSA), the membranes were incubated with the following primary antibodies: anti-CD63 (Abcam, United States) and anti-TSG101 (Abcam, United States). The membranes were then incubated with horseradish peroxidase (HRP)–conjugated secondary antibodies. Protein bands were visualized with an enhanced chemiluminescence detection kit and detected by an Amersham Imager 600 (GE, United States).

### 2.11. Statistical Analysis

GraphPad Prism software (version 9) was used for all statistical analysis. Data are expressed as the mean ± standard deviation (SD). Student's *t*-test was used to compare data between the two groups. *p* < 0.05 was considered statistically significant.

## 3. Results

### 3.1. Initiation of Perimenopause

**Figure 2:**
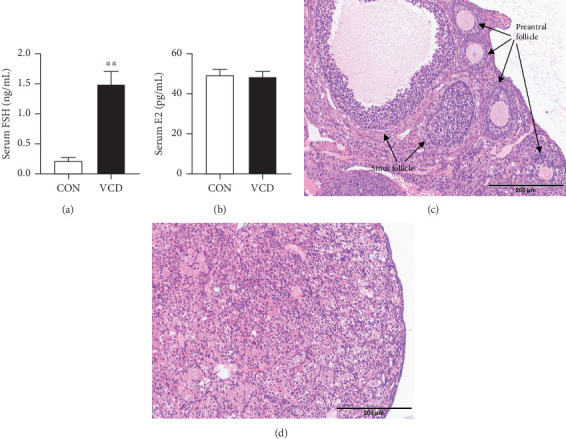
Plasma hormone changes in mice during the perimenopausal period at the end of the study. (a) Serum FSH levels. (b) Serum E2 levels. (c, d) H&E staining of ovaries (200×) in the CON (c) and VCD (d) groups. Values are presented using mean ± SD. ⁣^∗∗^*p* < 0.01 versus CON; *n* = 10 for each group.

To study the effect of perimenopause on the development of impaired insulin sensitivity, female C57BL/6 mice were treated with VCD or vehicle and fed a HFD. Mice in the VCD group (HFD/VCD) were treated with VCD for 20 consecutive days to cause ovarian failure, which is analogous to menopause in humans, and those in the control group (HFD) were injected with sesame oil. Plasma FSH levels were higher (*p* < 0.05) in the VCD group compared to the control group, and plasma E2 levels were not significantly different between the two groups (Figures 2(a) and 2(b)). Histological analysis indicated that VCD-treated ovaries had no ovarian follicles compared to CONs, which contained follicles at all stages of development and corpora lutea (Figures 2(c) and 2(d)).

### 3.2. Impaired Insulin Sensitivity in Perimenopausal Mice

**Figure 3:**
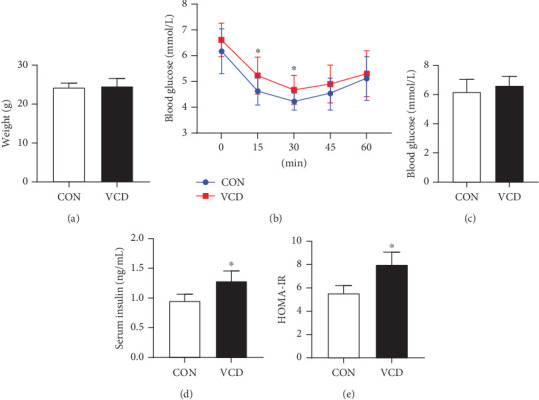
Effects of perimenopause on body weight, blood glucose, and insulin resistance in CON and VCD mice. (a) Body weight in the CON and VCD groups. (b) ITT in the CON and VCD groups. (c) Fasting blood glucose levels. (d) Serum insulin levels. (e) HOMA-IR scores. Values are presented as the mean ± SD. ⁣^∗^*p* < 0.05 versus CON; *n* = 10 for each group.

The body weights at Day 52 after VCD injection were not significantly different between the two groups (Figure 3(a)). ITT and HOMA-IR were utilized to assess the status of insulin resistance. The ITT results showed that after 17 days of HFD treatment, glucose levels were significantly increased at 15 and 30 min in perimenopausal mice (*p* < 0.05) after ip injection of insulin (Figure 3(b)). There was no significant difference in fasting blood glucose levels between the HFD/VCD and the HFD groups, but mice in the HFD/VCD group had a higher fasting insulin level compared to those in the HFD group. Subsequently, the HOMA-IR score in the VCD-treated group was significantly increased compared to that in the control group, which suggested that perimenopausal mice had abnormal insulin sensitivity (Figures 3(c), 3(d), and 3(e)).

### 3.3. Decreased Hepatic Glycogen Content During Perimenopause

**Figure 4:**
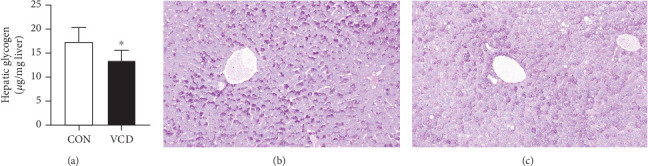
Hepatic glycogen content changes during the perimenopausal period at the end of the study. (a) Hepatic glycogen content. (b, c) PAS staining of liver sections showed that the glycogen in VCD mice (c) was decreased compared to that in CON mice (b). Red indicates glycogen, and purple indicates nuclei of liver cells. Values are presented as the mean ± SD. ⁣^∗^*p* < 0.05 versus CON; *n* = 5 for each group.

Compared to the control group, the liver glycogen content was reduced in the HFD/VCD group (*p* < 0.05) (Figure 4(a)). Similarly, PAS staining of liver sections showed decreased glycogen content in HFD/VCD mice with the development of ovarian failure at the end of the study (Figures 4(b) and 4(c)).

### 3.4. Extraction and Characterization of Mouse Plasma Exosomes

**Figure 5:**
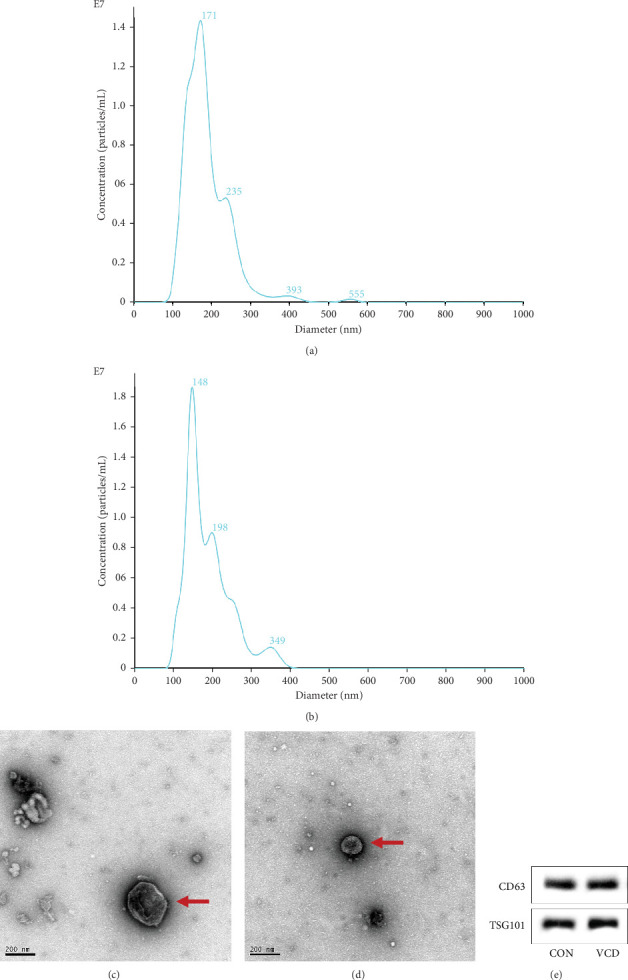
Characterization of exosomes purified from plasma of CON and VCD mice. (a, b) NTA of exosomes from plasma in CON (a) and VCD (b) groups. (c, d) TEM images of exosomes (red arrow) from CON (c) and VCD (d) groups. Scale bar = 200 nm. (e) Expression of the CD63 and TSG101 exosomal markers was confirmed by Western blot analysis in the CON and VCD groups.

Plasma exosomes were characterized using NTA and TEM, which demonstrated that more than 70% of the particles were 30–200 nm in diameter (Figures 5(a), 5(b), 5(c), and 5(d)) in both groups. Western blot analysis detected two typical exosome makers, namely, CD63 and TSG101, in all vesicles in both groups (Figure 5(e)).

### 3.5. Analysis of Exosomal miRNA Alterations During Perimenopause

**Figure 6:**
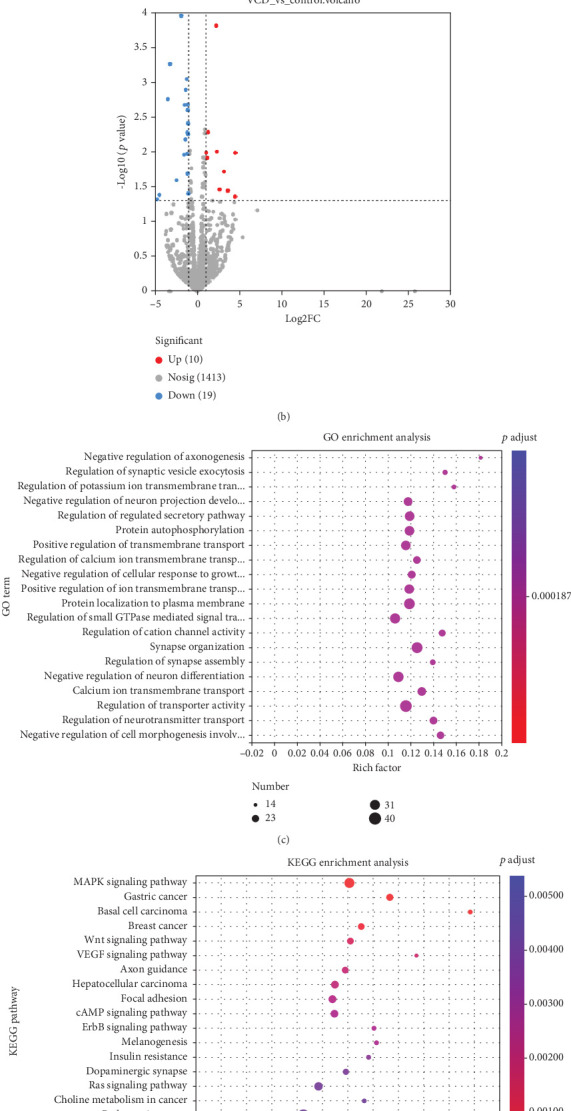
Analysis of DEMs and their target genes in plasma exosomes during perimenopause. Amount (a) and profiling (b) of miRNAs in plasma exosomes. (c, d) Distribution of GO classification (c) and KEGG pathway enrichment (d) among the predicted miRNA target genes during perimenopause.

Figure 6(a) shows that the isolated miRNA contents were similar in the CON and VCD groups. However, the exosomal miRNA profiles in perimenopausal mice were different from those in the control group. There were 29 significantly different miRNAs between the two groups, including 10 significantly upregulated DEMs and 19 significantly downregulated DEMs (|fold change| > 2.0, *p* < 0.05) (Figure 6(b)). Compared to the CON group, 3_26238 was the most upregulated miRNA (log2(fold change) = 4.49), whereas 12_9048 was the most downregulated miRNA (log2(fold change) = −4.76).

GO analysis indicated that the predicted DEM target genes were involved in regulation of synaptic vesicle exocytosis, protein autophosphorylation, protein localization to plasma membrane, and other related biological processes (Figure 6(c)). KEGG pathway analysis suggested that the DEM target genes were mainly involved in hepatocellular carcinoma, cyclic adenosine monophosphate (cAMP) signaling pathway, insulin resistance, and other related pathways (Figure 6(d)).

### 3.6. Altered mRNA and Establishment of the DEM–DEG Network

**Figure 7:**
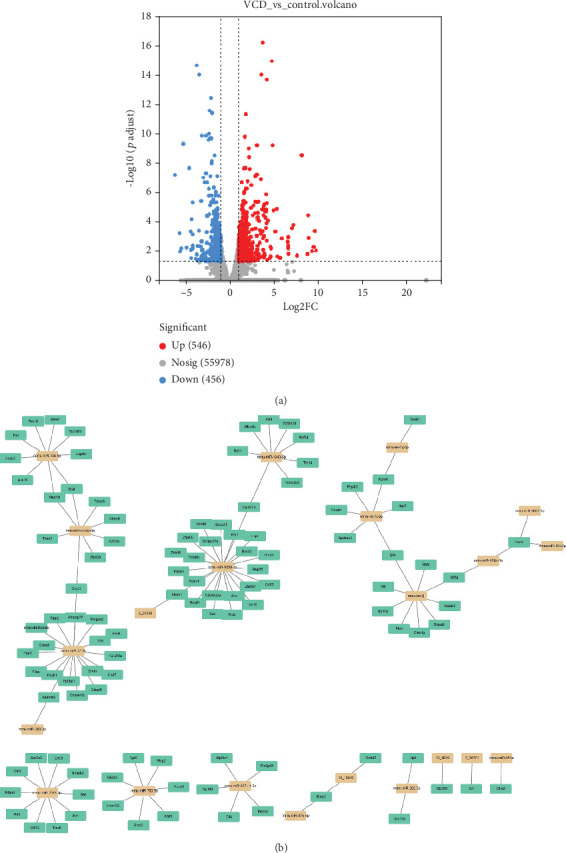
(a) mRNA expression profiling in hepatic tissue during perimenopause. (b) DEM–DEG regulatory network. The miRNA-mRNA network was constructed using Cytoscape 3.10 software following correlation analysis of predicted miRNA targets of DEGs. miRNAs are indicated by orange rectangles, and mRNAs are indicated by green rectangles.

The RNA-seq results showed that there were 1002 DEGs (|fold change| > 2.0, *p* < 0.05) between the VCD and CON groups, including 546 upregulated DEGs (fold change > 2.0 and *p* < 0.05) and 456 downregulated DEGs (fold change < −2.0, *p* < 0.05). *Alas2* and *Cyp3a44* had the highest mRNA expression (log2(fold change) = 3.73) and lowest mRNA expression (log2(fold change) = −3.73), respectively (Figure 7(a)). The DEM–DEG network was established based on the negative regulatory relationship, in which miR-17-3p, miR-134-5p, miR-700-5p, miR-6899-3p, *G6pdx*, *Ptpn2*, *Lepr*, *Kras*, and *Braf* were considered the important miRNAs and t-DEGs in the network (Figure 7(b)).

### 3.7. Validation of miRNAs and mRNAs by qRT-PCR

**Figure 8:**
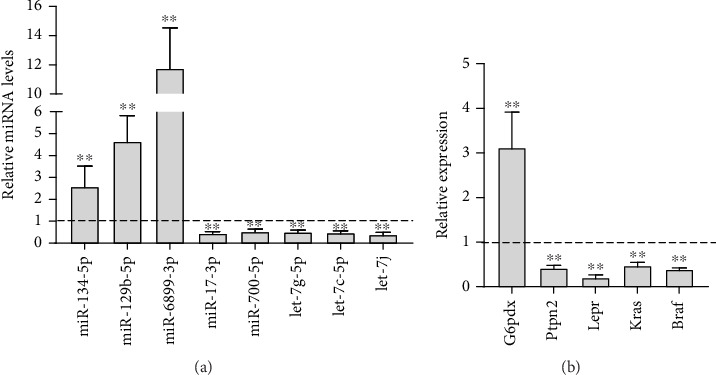
Validation of miRNA and mRNA expression of CON and VCD mice through real-time PCR. (a) Differential expression levels of miRNAs in plasma exosomes. (b) Differential expression levels of mRNAs in hepatic tissue during perimenopause. Data are shown as mean ± SD. ⁣^∗∗^*p* < 0.01 versus CON; *n* = 3 for each group.

The miRNA and mRNA RNA-seq results were validated by qRT-PCR (Figures 8(a) and 8(b)). In plasma exosomes, the expression levels of miR-134-5p, miR-129-5p, and miR-6899-3p were increased, whereas the expression levels of miR-17-3p, miR-700-5p, Let-7g-5p, Let-7c-5p, and Let-7j were decreased. The expression levels of these selected miRNAs were consistent with the RNA-seq results. Moreover, in the liver at the onset of impaired insulin action during perimenopause, the expression of *G6pdx* was increased, but the expression of*Ptpn2*, *Lepr*, *Kras*, and *Braf* was decreased.

## 4. Discussion

Perimenopause is defined as the transitional period that occurs before menopause. Typically, perimenopause begins when women are in their 40s, but some women may notice changes as early as their mid-30s. Due to irregular ovulation and abnormal ovarian function during the transitional phase, cycle lengths are variable, and estrogen levels fluctuate with a gradual decline until circulating E2 levels reach very low levels at menopause. In addition, FSH levels tend to increase during the transitional phase. Several studies have suggested that postmenopausal women have increased insulin resistance compared to premenopausal women, predisposing them to the development of diabetes [16, 30]. However, the relationship between perimenopause and insulin resistance is not yet fully understood, and the available data are contradictory. Therefore, the impact of perimenopause on the onset of impaired insulin response and insulin resistance needs to be further explored.

In the present study, VCD was used to induce gradual ovarian failure in mice. The VCD model preserved the period of irregular cycling and fluctuating hormones analogous to perimenopause (periovarian failure in mice) in human [28]. Compared to the CON group, the FSH level was significantly increased in the VCD group, while there was no difference in the E2 level between the two groups. VCD targets primary and primordial follicles, and it has no toxic effect on ovaries [31]. In the present study, ovarian tissues in the model mice were retained but showed no follicles. The hormone changes and histological analysis suggested that the VCD model mice closely approximate the natural progression of perimenopause in women. In addition, HFD is a well-established model of obesity and insulin resistance, even after a short term [32]. Therefore, VCD model mice were fed with a HFD during the last 17 days of the progressive perimenopause period. Although there was no difference in body weight between the CON and VCD groups after feeding a HFD for 17 days, mice in the VCD group developed higher fasting insulin levels and HOMA-IR than those in the CON group, suggesting the induction of abnormally increased insulin levels and insulin resistance together with the development of perimenopause. As impaired glycogen synthesis in the liver has been identified as a significant factor in insulin resistance, PAS staining was used to histologically evaluate the glycogen content in liver tissues in both groups. Consistent with the glycogen assay results, histological analysis showed a decrease in liver glycogen content in mice during perimenopause. These results suggested that impaired insulin action is likely to occur in women near menopause. However, the underlying biological mechanism of how perimenopause impacts insulin action remains unclear.

Recently, circulating miRNAs in extracellular vesicles (EVs) have been identified as important regulators of intercellular communication and signaling [33]. EVs may be involved in liver pathology because they control the expression of multiple genes in recipient cells [34]. Increasing evidence has shown that circulating miRNAs in EVs are critical posttranscriptional regulators of gene expression involved in metabolic changes accompanying the progression of obesity, insulin resistance, and metabolic disorders [35]. In the present study, changes in circulating miRNA levels may reflect defects in insulin response in the liver during perimenopause, suggesting that altered exosomal miRNAs and mRNAs may be related to the negative effects of periovarian failure on insulin resistance.

Circulating miRNAs within EVs have emerged as important regulators of intercellular communication and signaling, particularly in the context of liver pathology. These miRNAs play a crucial role in modulating gene expression in recipient cells, which is particularly relevant in metabolic disorders, such as obesity and insulin resistance [36, 37]. Recent studies have indicated that alterations in the levels of circulating miRNAs in EVs may reflect disruptions in insulin signaling pathways within the liver, especially during perimenopause. The present study posits that these changes in miRNA profiles are linked to the adverse effects of ovarian failure on insulin resistance. Specifically, the dysregulation of exosomal miRNAs and mRNAs may contribute to the pathophysiological processes underlying liver dysfunction [38, 39]. The relationship between circulating miRNAs in EVs and liver pathology is underscored by their ability to influence various signaling pathways that regulate metabolic functions. For instance, miRNAs encapsulated in EVs can be transferred to liver cells, where they modulate gene expression and impact metabolic homeostasis. This intercellular communication mechanism highlights the potential of circulating miRNAs as biomarkers for liver disease and as therapeutic targets for metabolic disorders [40].

According to the RNA-seq data, there were 29 DEMs between the CON and VCD groups based on a cutoff of > 2.0-fold change in expression. GO and KEGG pathway analysis indicated that the DEM target genes were mainly involved in protein localization to plasma membrane, cAMP signaling pathway, insulin resistance, and other biological processes or pathways. In addition, the expression changes of 1002 DEGs were also analyzed. To further explore the exosomal miRNAs and their target mRNAs expressed within the liver, a miRNA-gene network based on the negative relationship was constructed to determine the direct interaction between the DEMs and DEGs. In the network, miR-17-3p, miR-134-5p, miR-700-5p, miR-6899-3p, *G6pdx*, *Ptpn2*, *Lepr*, *Kras*, and *Braf* were considered the key miRNAs and genes. Based on their biological functions, glucose-6-phosphate dehydrogenase (G6PD) is an enzyme encoded by the *G6PD* gene, which is located on the X chromosome. G6PD deficiency is an X-linked recessive disorder that affects the activity of the G6PD enzyme [41]. In the present study, G6PD was predicted to be regulated by miR-700-5p, which may protect against insulin resistance, while G6PD upregulation may lead to oxidative stress and functional defects in the liver, heart, and pancreatic *β*-cells [42–44]. Additionally, G6PD plays a role in adipogenesis and glucose uptake. G6PD deficiency may protect against the development of insulin resistance and a prediabetic state [45–47]. The leptin receptor (LEPR), which was predicted to be the target of miR-6899-3p, plays a significant role in liver insulin resistance. Leptin resistance and impaired LEPR signaling contribute to the development of hepatic insulin resistance and are associated with several conditions, such as nonalcoholic fatty liver disease (NAFLD) [48, 49]. Thus, the DEM–DEG network should be utilized to perform further research.

To confirm the RNA-seq results, qRT-PCR was used to validate eight miRNAs and five mRNAs. miR-134-5p, miR-129-5p, miR-6899-3p, miR-17-3p, miR-700-5p, Let-7g-5p, Let-7c-5p, and Let-7j were selected as a result of their correlation with insulin resistance, forkhead box O1 (FOXO1) signaling, or phosphoinositide 3-kinase (PI3K)/protein kinase B (AKT) signaling [50–53]. *G6pdx*, *Ptpn2*, *Lepr*, and *Kras*, which are involved in insulin resistance, are regulated by different miRNAs [54, 55]. The qRT-PCR results were consistent with the sequencing results.

Insulin signaling in the liver tissue is essential for the regulation of glucose and lipid metabolism. The insulin signaling pathway involves the activation of the insulin receptor (IR) by insulin binding, leading to the activation of downstream signaling molecules. One of the key downstream pathways is the PI3K/AKT pathway [56]. Insulin activates PI3K, which in turn activates AKT. AKT phosphorylates various substrates, including FOXO1, leading to its degradation and reduced expression of gluconeogenic genes [57, 58]. Several lines of evidence have demonstrated that the impairment of this biological process has adverse effects on hepatic insulin resistance, increasing the risk of diabetes or other metabolic disease [59]. G6PD is an enzyme that generates nicotinamide adenine dinucleotide phosphate (NADPH) and ribose-5-phosphate for biosynthetic processes. G6PD deficiency impacts insulin signaling activation, while LEPR and KRAS modulate insulin sensitivity and glucose metabolism [44, 60, 61]. Protein tyrosine phosphatase nonreceptor Type 2 (PTPN2) is a tyrosine phosphatase that regulates insulin signaling by inactivating the IR through dephosphorylation of the IR *β*-chain [61]. In addition, BRAF, as a serine/threonine kinase, modulates the activity of the AKT pathway to regulate insulin-stimulated glucose uptake [62]. In the present study, aberrant expression of plasma exosomal miR-17-3p, miR-134-5p, miR-700-5p, and miR-6899-3p was predicted to target *Ptpn2*, *Braf*, *G6pdx*, *Kras*, and *Lepr*, respectively, which are involved in the regulation of insulin signaling in the liver during perimenopause. Thus, impairment of the insulin signaling response in the liver may be induced by these DEGs.

The present study elucidates the impaired insulin sensitivity associated with the progression of perimenopause and highlights the correlation between plasma exosomal miRNAs and the onset of insulin resistance. The findings indicate that exosomal miRNAs may carry abnormal cargos that influence gene expression in the liver, subsequently leading to dysregulated insulin responses. This underscores the potential role of exosomal miRNAs as mediators in metabolic disturbances during perimenopause. However, several aspects warrant further investigation to fully comprehend the mechanisms at play. First, while this study establishes correlations between specific miRNAs and DEGs involved in insulin resistance, it does not provide definitive evidence of causation. For instance, although miR-700-5p is predicted to regulate G6PD, additional functional studies are necessary to confirm this interaction and its biological implications. Without such experimental validation, it remains uncertain whether these miRNAs directly modulate insulin signaling pathways or if their alterations are merely associated with other underlying processes. Moreover, the validation of miRNAs and mRNAs through qRT-PCR was limited to a small subset—eight miRNAs and five mRNAs. This raises concerns regarding the generalizability of the findings across all identified DEMs and DEGs. Broader validation across diverse biological contexts is essential to reinforce the conclusions drawn from this research. Another critical limitation pertains to the lack of verification of results in human subjects. While animal models can provide valuable insights, they may not fully replicate human physiology or disease states. Therefore, further studies should aim to validate these findings in larger human cohorts to enhance their clinical relevance. Additionally, understanding the specific tissues from which these exosomal miRNAs originate is crucial for elucidating their roles in insulin signaling and liver function. Identifying the source tissues will facilitate a more comprehensive understanding of how these exosomal miRNAs interact within various biological systems and contribute to metabolic dysregulation. The study also highlights the need for future research to explore how altered expression of miRNAs and mRNAs regulates each other within the context of insulin signaling pathways. A deeper understanding of these molecular mechanisms will be vital for developing targeted therapeutic strategies aimed at mitigating insulin resistance during perimenopause.

In summary, while this study provides foundational insights into the role of plasma exosomal miRNAs in insulin resistance during perimenopause, addressing these limitations through extensive validation studies and functional analyses will be critical for advancing our understanding of metabolic disorders in women. Future investigations should focus on identifying serological biomarkers derived from plasma exosomes that could serve as measurable indicators of disease progression, ultimately aiding in the prevention and management of diabetes mellitus in this population.

## Figures and Tables

**Table 1:** Primer used for RT-PCR.

**Target gene**	**Primer sequence (5**⁣′**-3**⁣′**)**		**Size (bp)**
	**Forward**	**Reverse**	
*G6pdx*	GCTTGGACCGCCATTTTGT	GGCCCTGAAACCCTACAATGA	108
*Ptpn2*	AGAAAGGCTACGACGGCTCA	TGAAAAAGCAGTGTCCAGCCA	164
*Lepr*	CTGAGCCCAAAAACTGCGTC	GGAGTCAGGAAGGACACACG	152
*Kras*	GCGCCTTGACGATACAGCTAA	TACACAAAGAAAGCCCTCCCC	194
*Braf*	TGCTCAGGTCCCTTCATTTGT	TCAATTACATAGCACTTTTTGGGG	217
*GAPDH*	CATCAAGAAGGTGGTGAAGC	CATCGAAGGTGGAAGAGTGGG	119

## Data Availability

The original contributions presented in the study are included in the article. Further inquiries can be directed to the corresponding author.
